# Hiatal reconstruction is safe and effective for control of reflux after laparoscopic sleeve gastrectomy

**DOI:** 10.1186/s12893-022-01800-y

**Published:** 2022-09-21

**Authors:** Ben Indja, Daniel L. Chan, Michael L. Talbot

**Affiliations:** 1 Faculty of Medicine and Health, The University of Sydney, Sydney, NSW Australia; 2 Department of Surgery, St George Hospital, Sydney, NSW Australia; 3 Department of Surgery, Faculty of Medicine, The University of New South Wales, Sydney, NSW Australia; 4 Upper GI Surgery, St George Private Hospital, Suite 3, Level 5, 1 South, Sydney, NSW 2217 Australia

**Keywords:** Gastroesophageal reflux, Hiatal hernia, Laparoscopic sleeve gastrectomy, Bariatric surgery complications

## Abstract

**Background:**

Gastroesophageal reflux is a known complication following laparoscopic sleeve gastrectomy (LSG) as anatomical changes predispose to reduced lower esophageal sphincter pressure and development of hiatus hernia. The mainstay of surgical management has been Roux-en-Y gastric bypass (RYGB) which is not without risk. Hiatus hernia repair (HHR) with surgical reattachment of the oesophagus to the crura, recreating the phreno-esophageal ligament is a simple procedure specifically targeting a number of anatomical changes responsible for reflux in this population.

**Methods:**

We conducted a single centre retrospective analysis of adult patients with post-sleeve reflux refractory to medical treatment, managed with either HHR, RYGB or One-anastomosis Duodenal switch (OADS). PPI use and symptoms of reflux were assessed at early and mid-term time points via validated questionnaires.

**Results:**

99 patients were included, of these the surgical procedure was HHR alone in 58, RYGB in 29 and OADS in 12. At early follow-up control of reflux symptoms was achieved in 72.4% after HHR, 82.1% after RYGB and 100% after OADS with no significant difference between groups (p = 0.09). At mid-term followup (median 10 months IQR 7–21) there was no significant difference in the presence of symptomatic reflux as determined by post-op Visick score nor a difference in PPI use. The GerdQ score was significantly lower after OADS as compared to HHR and RYGB (4.6 ± 2.3 vs 7.7 ± 2.2 vs 8.7 ± 3.5, p = 0.006).

**Conclusion:**

HHR with reconstruction of the phreno-esophageal ligament is a safe and effective procedure for patients with reflux after LSG, that avoids more complex operations such as RYGB and OADS and their associated long-term sequelae.

## Introduction

Laparoscopic sleeve gastrectomy (LSG) is the most common bariatric procedure performed worldwide [1] and demonstrates excellent results in reducing weight and obesity-related morbidity [2, 3]. The early outcomes of LSG are on par with Roux-en-Y gastric bypass (RYGB) [4], and its popularity is driven by comparatively reduced perioperative complications by being a less technically demanding procedure and the reduced risk of malnutrition and less requirement for strict nutritional surveillance and supplementation [5, 6].

One of the most common complications of LSG is gastro-esophageal reflux disese (GERD). The incidence of symptomatic reflux has been reported as high as 57% within 10 years of surgery and this has been shown to have a negative impact on quality of life and to increase the risk of Barrett’s esophagus [7]. De novo hiatus hernias seem to have a strong correlation with the development of reflux after LSG [7, 8]. Patients who have no or minimal reflux soon after surgery can develop progressive reflux symptoms years after surgery despite achieving and maintaining clinically significant weight loss.

The lower esophageal sphincter (LES) plays a key role in preventing gastric contents refluxing up into the esophagus. There are three components of the LES that contribute towards this anti-reflux mechanism. Firstly, the intrinsic sphincter consists of smooth muscle fibres that remain tonically contracted and provide a baseline pressure differential between the stomach and esophagus [9]. The second component is the external crura-these skeletal muscle fibres extending from the diaphragm are superimposed with those fibres of the intrinsic sphincter [10]. The external crura ensure maintenance of the LES pressure at times when the pressure differential may favor movement of contents from the stomach up into the esophagus such as during inspiration or valsalva. The third contributor to the LES is the phreno-esophageal ligament which holds the LES complex together. Fibres of the phreno-esophageal ligament originate from both the abdominal and thoracic surfaces of the diaphragm, inserting into the esophageal adventitia [11].

**Fig. 1:**
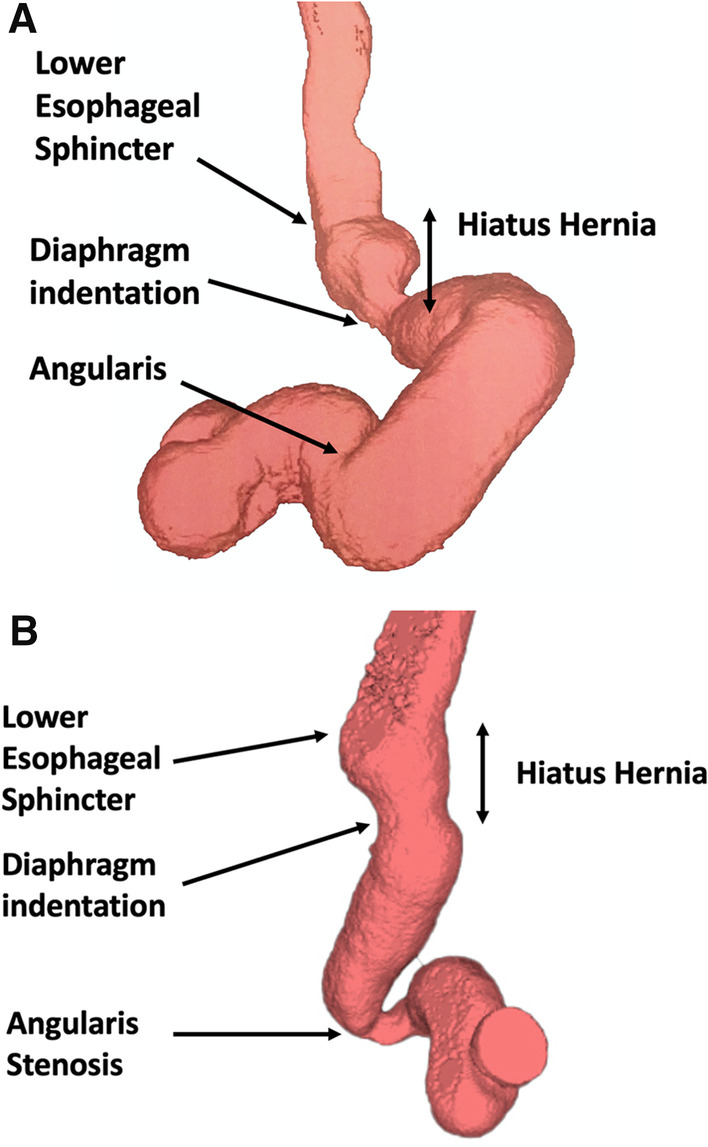
**a** 3D CT scan post sleeve gastrectomy demonstrating hiatal hernia. **b** 3D CT scan post sleeve gastrectomy demonstrating small hiatal hernia and angularis stenosis

Anatomical changes during LSG impair normal physiology and lead to the development of reflux. The shape of the gastric tube and loss of the gastric fundus predispose to telescoping of the stomach into the chest (Fig. 1a) [12]. The phreno-esophageal ligament is often disrupted during LSG thus resulting in weakening of the structural framework between the intrinsic sphincter and external crura. Separation of these two components which occurs with hiatal hernia results in a hypotensive LES. The reduced volume of the gastric tube also results in higher intragastric pressures, which the impaired LES is less likely to be able to withstand [13]. Sleeve morphology also has a role in post-LSG reflux, with incisura stenosis (Fig. 1b) and/or proximal tube dilation being a likely, albeit poorly defined driver of reflux and regurgitation in some patients.

In approaching the management of intractable reflux after LSG, the default strategy has been conversion to RYGB. This is due to the noticeable improvement in symptoms of GERD and reduction in use of PPIs seen in patients who have undergone RYGB for obesity [14], however reflux symptoms can return in up to half of these patients in longer term follow-up [15]. For patients who do not require additional weight loss, RYGB is not an insignificant undertaking-revision bariatric surgery comes with increased risks of anastomotic leak, bleeding and reoperation [16], along with long term risks specific to RYGB such as internal hernias, marginal ulceration and nutritional deficiencies [17]—all of which often play a role in the decision for LSG over RYGB when selecting a primary weight loss procedure. Conversion of LSG to RYGB doesn’t seem to guarantee reflux control with many patients still taking PPI in short term follow-up [18]. Additionally the literature present minimal objective data, with the majority of LSG to RYGB studies failing to report PPI use or symptom scores [19, 20]. The sole study looking at objective data and post-conversion analysis saw persisting reflux symptoms in nearly a third of patients converted to RYGB from LSG, with persisting abnormal esophageal acid exposure despite normal hiatal anatomy and manometry post-conversion [21].

We hypothesize that a more simplified surgical approach involving hiatal hernia repair with cruroplasty and phreno-esophageal ligament repair or esophagopexy (HHR) would sufficiently address the anatomical changes that result in reflux after LSG in patients who lack anatomic contraindications to such surgery and who do not require further weight-loss, or otherwise prefer to avoid RYGB. The aim of this study was to compare reflux outcomes in patients with previous LSG who subsequently underwent HHR, RYGB or one-anastomosis duodenal switch (OADS) for medically refractory reflux.

## Methods

### Participants

**Fig. 2:**
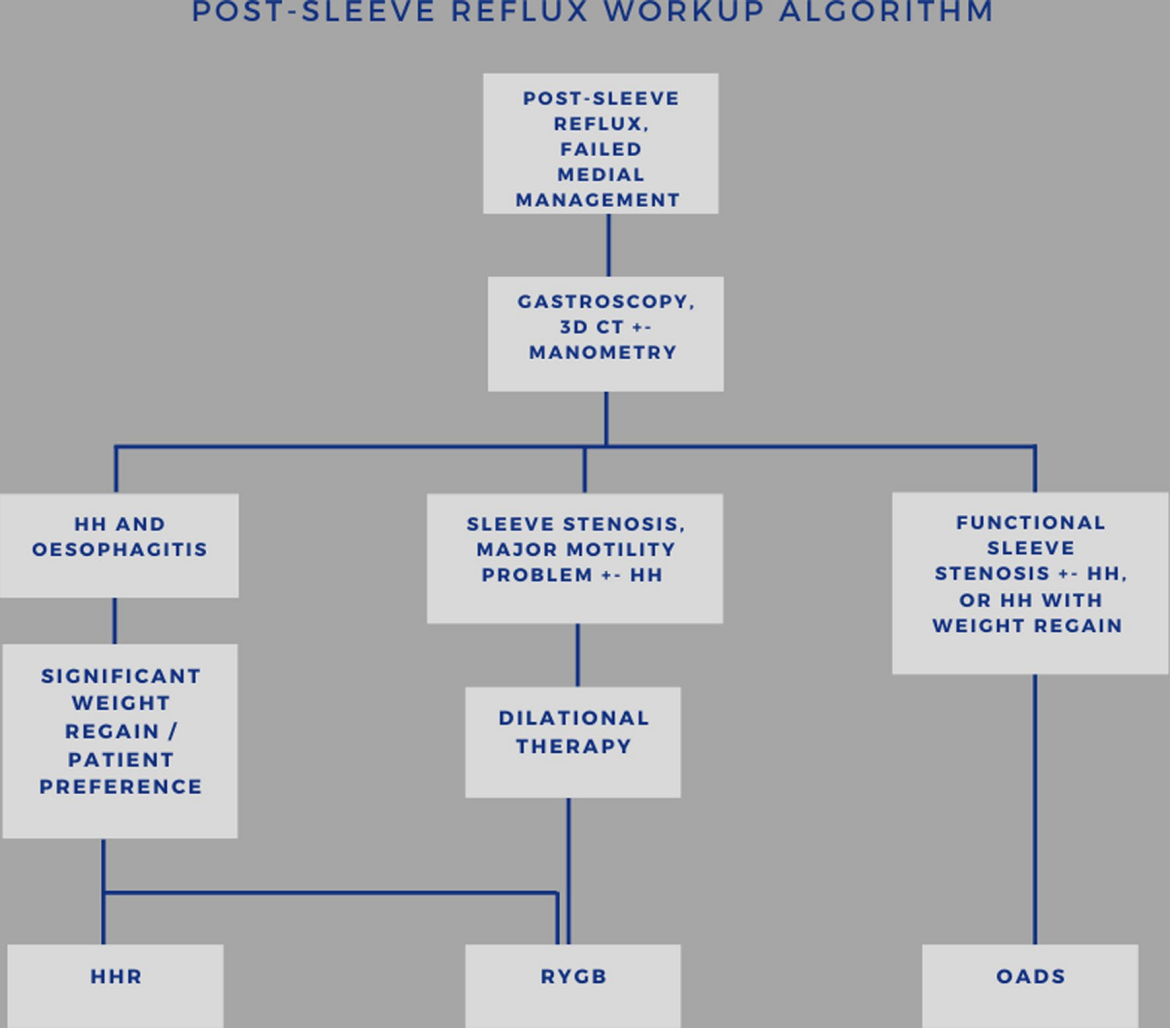
Post-sleeve reflux algorithm to guide workup after failure of medical therapy. *HH* hiatus hernia, *HHR* hiatus hernia repair, *RYGB* Roux-en-Y gastric bypass, *OADS* one-anastomosis duodenal switch

This is a retrospective cohort study involving adult patients (> 18 years) with previous LSG who underwent revision surgery for medically refractory reflux. Retrospective chart review of a single high volume bariatric centre was performed. Inclusion criteria included (1) LSG performed between 2010 and 2020 (2) Failure of maximal medical therapy (twice daily proton pump inhibitors (PPIs) lifestyle and dietary advice) requiring subsequent surgical intervention. All patients underwent workup which included gastroscopy and/or one of a combination of 3D functional computerized tomography, manometry and esophogeal pH studies to assist in determining the most suitable surgical approach. An example of the workup algorithm we used as a guide is demonstrated in Fig. 2. Retrospectively identified patients were recruited to participate at mid-term follow-up which involved completing two validated reflux symptom questionnaires, either online or via telephone.

### Outcomes and follow-up

Early assessment occurred at first post-operative clinic follow-up within 30 days. The primary outcome at this point was the presence of gastroesophageal reflux symptoms (heartburn, dysphagia, regurgitation) and PPI use. Patients were categorized into four separate groups depending on the type of resolution at follow-up which included (1) symptom free without ongoing PPI therapy, (2) Occasional symptoms but well controlled with breakthrough PPI use, (3) Symptoms controlled with ongoing reliance on regular PPI therapy and (4) Breakthrough reflux symptoms while taking PPI therapy. For midterm follow-up patients were contacted between January 2021 and March 2021 to participate in phone or online surveys. The primary outcomes included the GERDQ questionnaire and a Post-op Visick score along with PPI use. Secondary outcomes included perioperative mortality and morbidity (30 days) along with reinterventions for either operative complications or persistence of reflux symptoms.

### Reflux questionnaires

The Post-op Visick score is a simple validated method to assess the efficacy of surgical intervention for symptoms of GERD [22]. In particular it has been shown to accurately reflect patient quality of life after anti-reflux surgery and address the main symptom of reflux-heartburn [22, 23]. The post-op Visick score simply asks patients to select one of four statements that best describes their symptoms of reflux—(1) No symptoms, resolved, (2) Mild occasional symptoms not requiring treatment, (3) Frequent symptoms requiring regular or daily treatment, (4) severe or daily symptoms despite daily medical treatment.

The second survey participants completed was the GerdQ questionnaire which has been demonstrated to be a useful non-invasive tool in the diagnosis of reflux and in the implementation of further investigative or management strategies [24, 25]. The questionnaire consists of four questions related to reflux symptoms and two questions relating to the impact of reflux symptoms on quality of life, specifically impact on sleep and requirement for additional medications. Patients are asked to indicate the frequency for each question that relates to the previous seven days (Table 1). A score less than 8 suggests a low probability of reflux, while a score 8 or higher suggests a high probability of reflux [26]. The possible scores range from 0 to 18.

**Table 1:** GerdQ questionnaire template

Question	Frequency score (points) for symptoms			
	0 day	1 day	2–3 days	4–7 days
1. How often did you have a burning feeling behind your breastbone (Heartburn)?	0	1	2	3
2. How often did you have stomach contents (liquids or food) moving upwards to your throat or mouth (regurgitation)?	0	1	2	3
3. How often did you have pain in the centre of the upper stomach?	3	2	1	0
4. How often did you have nausea?	3	2	1	0
5. How often did you have difficulty getting a good nights sleep because of your heartburn and / or regurgitation?	0	1	2	3
6. How often did you take additional medication for your heartburn and / or regurgitation, other than what your doctor told you to take (e.g. Gaviscon, Rennie)	0	1	2	3

### Surgical approach

The decision of which anti-reflux operation to perform was determined by the treating surgeon in accordance with specific patient preferences and additional indications such as weight regain, sleeve stenosis or dilation of the gastric tube (Fig. 2). For laparoscopic HHR the hiatus hernia was dissected out and reduced followed by posterior cruroplasty and esophagopexy whereby small bites of esophagus and crura are taken with a non-absorbable suture from 6 o’clock to the 11 o'clock position in an anticlockwise direction, holding 1–2 cm of esophagus inside the abdominal cavity. Reinforcement of hiatus hernia repair alone was with light microporous mesh fashioned in a “V” shape to support the cural repair, while for RYGB or OADS no mesh was used. For laparoscopic RYGB the hiatus was dissected and repaired as above and a transected lesser curvature pouch was created over a 38 fr bougie with the majority of cases banded with placement of a silastic ring at 7.5–8 cm. An antecolic divided omega Roux loop was raised for gastroenterostomy and an enteroenterostomy followed by internal defect closure. OADS was performed by initially repairing any hiatus hernia and mobilizing the distal part of gastric tube and post-pyloric duodenum. The small bowel was then measured and divided 330 cm proximal to the terminal ileum for the common limb. The duodenum was then divided 1 cm beyond the pylorus with division of the right gastric artery to allow the tube to straighten out into the midline, followed by a handsewn pyloroenterostomy anastomosis.

### Statistical analyses

Baseline characteristics are presented as a raw percentage (%) or for continuous variables as mean and standard deviation. Comparison of categorial variables was performed using Fisher’s Exact test (when values < 5) or Chi Squared test and comparison of means with ANOVA. A p-value < 0.05 was considered to be significant. All statistical analyses were performed using STATA 16 (StataCorp. 2019. Stata Statistical Software: Release 16. College Station, TX: StataCorp LLC).

This study had ethics approval (Approval number HC200283) from the University of New South Wales.

## Results

There were 99 patients included in the study who underwent surgical management of reflux after previous LSG. The procedures included HHR in 59%, RYGB in 29% and OADS in 12%. 11 patients subsequently underwent a redo anti-reflux procedure due to persistent or delayed return of reflux symptoms. Two patients proceeded to HHR after RYGB; One patient after OADS was converted to RYGB; In the HHR group, four patients were converted to RYGB, three to OADS and one had redo HHR.

The mean age of patients at time of LSG was 43 ± 11 years with the majority female, accounting for 82%. There were no significant differences in baseline characteristics between each group, including the presence of reflux prior to LSG (Table 2). The mean time between LSG and anti-reflux procedure was 63.8 ± 37.6 months.

**Table 2:** Baseline characteristics at time of laparoscopic sleeve gastrectomy

	HHR % (n = 58)	RYGB % (n = 29)	OADS % (n = 12)	p-value
Females	84.5 (49)	79.3 (23)	75 (9)	0.6
Age	43.1 + -11.1	44.9 + -11.1	39.9 + -10.4	0.43
Smoker	5.66 (3)	15.4 ()	25 (3)	0.25
Ex-smoker	15.1 (8)	11.5 (3)	16.7 (2)	0.25
HTN	35.2 (19)	27 (7)	25 (3)	0.77
Hypercholesterolemia	16.7 (9)	19.2 (5)	25 (3)	0.75
Type 2 diabetes	16.7 (9)	7.7 (2)	0	0.3
Insulin resistance	14.8 (8)	24 (6)	33.3 (4)	0.28
Sleep apnoea	18 (9)	18.2 (4)	20 (2)	1
Prior reflux	29.6 (16)	32.1 (9)	18.2 (2)	0.71

*HHR* hiatus hernia repair, *RYGB* Roux-en-Y gastric bypass, *OADS* one-anastomosis duodenal switch, *PPI* proton pump inhibitors, *HTN* hypertension

Table 3 demonstrates the primary anatomical findings based on pre-operative 3D-CT or endoscopy.

**Table 3:** Primary anatomical findings determined by 3D-CT or endoscopy

	HHR % (n = 58)	RYGB % (n = 29)	OADS (n = 12)
Hiatus hernia	72 (42)	34 (10)	33.3 (4)
Sleeve stenosis	1.7 (1)	21 (6)	33.3 (4)
Dilated proximal gastric tube	7 (4)	14 (4)	33.3 (4)
Nil abnormality detected	19 (11)	31 (9)	0

*HHR* hiatus hernia repair, *RYGB* Roux-en-Y gastric bypass, *OADS* one-anastomosis duodenal switch, *PPI* proton pump inhibitors, *HTN* hypertension

There were no perioperative deaths. Two patients in the RYGB group had early post-operative gastrointestinal bleeds requiring diagnostic endoscopy. Both were related to the proximal anastomosis, however only one required endoscopic intervention with clipping of a vessel. One patient in the RYGB group was converted to laparotomy due to insufficient space in the peritoneal cavity after insufflation. One patient in the OADS group required a revision procedure with lengthening of the common channel due to persistent profuse diarrhea. There were no perioperative complications in the HHR group.

**Fig. 3:**
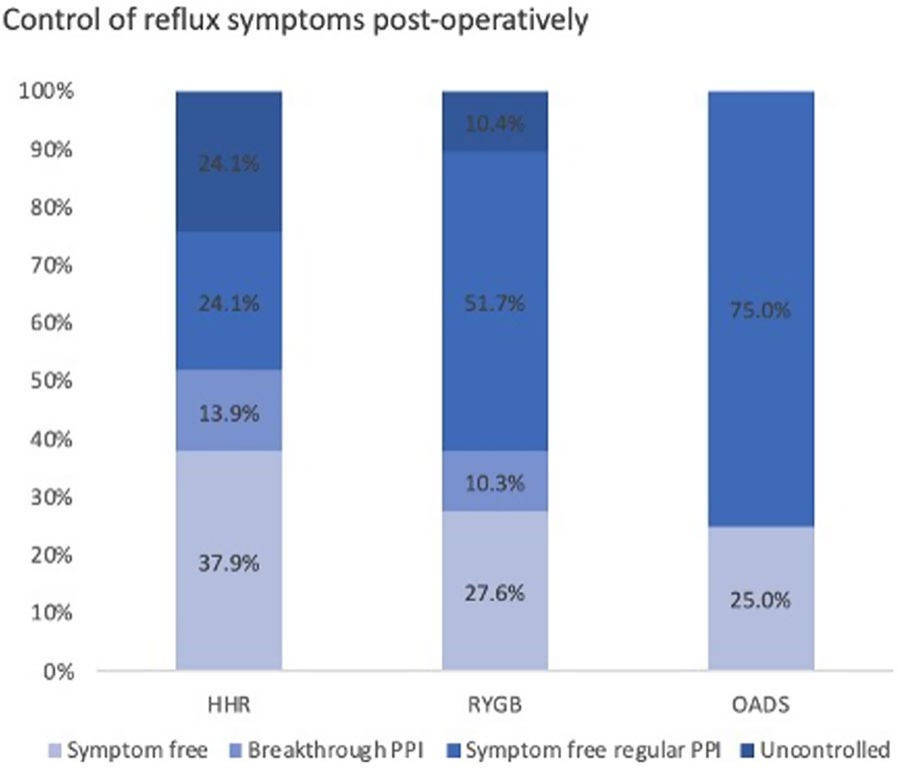
PPI requirement and early control of reflux at early follow-up. *HHR* hiatus hernia repair, *RYGB* Roux-en-Y gastric bypass, *OADS* one-anastomosis duodenal switch

At early follow-up the percentage of patients who reported adequate control of reflux symptoms, with or without an ongoing requirement for PPI therapy was 72.4% of patients in the HHR group, 82.1% after RYGB and 100% after OADS with no significant difference demonstrated between each group (p = 0.09) (Table 4). Figure 3 demonstrates the degree of symptom control based of the four early primary outcome measures. The proportion of patients who had ceased PPI was 38% for HHR, 28% for RYGB and 25% for OADS, with no significant difference between groups (p = 0.6).

**Table 4:** Control of reflux symptoms at early follow-up after anti-reflux procedure

	Reflux symptom free % (n)	Symptomatic reflux % (n)
HHR (58)	72.4 (42)	27.6 (16)
RYGB (29)	82.1 (23)	17.9 (5)
OADS (12)	100 (12)	0
Fisher’s exact = 0.09		

*HHR* hiatus hernia repair, *RYGB* Roux-en-Y gastric bypass, *OADS* one-anastomosis duodenal switch

A total of 43 patients completed mid-term follow-up symptom surveys at a minimum of 6 months (mean 18.5 ± 18.7 months) with a mean age of 43.8 ± 10.6. 37, (90%) were female. A total of 24 underwent HHR, 11 RYGB and 7 OADS.

The mean post-op Visick score was 2.17 ± 0.99, with no significant difference demonstrated between groups (Table 5).

**Table 5:** Late outcomes of reflux symptoms and PPI use after surgical management of reflux after laparoscopic sleeve gastrectomy

	HHR (25)	RYGB (11)	OADS (7)	p-value
Visick score	2.2 ± 0.9	2.2 ± 1.2	1.85 ± 1.2	0.72
GerdQ	7.7 ± 2.2	8.7 ± 3.5	4.6 ± 2.3	0.006
PPI use (%)	45.5	45.8	57.2	0.88

*HHR* hiatus hernia repair, *RYGB* Roux-en-Y gastric bypass, *OADS* one-anastomosis duodenal switch, *PPI* proton pump inhibitors

The GerdQ scores are shown in Table 5. There was a significant difference between groups (F = 5.83, p = 0.006), however this was driven by the OADS group. In subgroup analysis there was no difference in GerdQ scores between the HHR and RYGB groups (p = 0.36).

Ongoing daily use of PPI was similar between groups being 45.8% (HHR), 45.5% (RYGB) and 57.2% (OADS).

## Discussion

De novo gastroesophageal reflux is seen frequently in long term follow-up after LSG [27], however the published literature is heterogeneous regarding the procedures refluxogenic potential. While early development of reflux has been reported as a frequent early post-op complication by some authors [28–30], others have shown that clinical reflux can be uncommon after LSG when combined with routine hiatal repair [31]. Objective pH and manometry data pre and post LSG with liberal use of hiatal reconstruction also show that lower esophageal sphincter function and esophageal acid exposure can remain unchanged pre-and post-operatively [32]. As telescoping of the sleeve tube in the years after surgery is frequently observed and is strongly associated with the development of reflux symptoms [33], the concept that a patient who has developed reflux symptoms some years after LSG may be adequately treated by correcting this acquired anatomic abnormality is worthy of consideration.

While RYGB is an effective procedure to control symptoms of reflux after LSG, this study demonstrates that HHR is an alternate approach that could be considered as first line surgical management in appropriately selected patients. It is a reasonably simple procedure that allows maintenance of the sleeve anatomy and aims to directly address a dominant underlying cause of reflux after sleeve, that is, the disruption of the LES complex. It provides symptomatic outcomes non-inferior to those achieved with RYGB and OADS at both early and midterm follow-up.

Surgical reinforcement of the disrupted LES to address severe reflux is not a new idea. In 1964 Rampal described his technique of ligamentum teres cardiopexy (LTC), whereby the ligamentum teres is mobilised off the liver and anterior abdominal wall and slung and secured around the gastro-esophageal junction [34]. The technique has re-surfaced for post-sleeve reflux as a RYGB alternative [35, 36]. In this study cohort, phreno-esophageal ligament reconstruction by esophagopexy with a non-absorbable suture is analogous to the ligamentum teres approach and seems to deliver similar reflux control.

Our findings demonstrate that all three surgical approaches are very safe. The HHR group had no significant early complications, compared to three in the RYGB group and one in the OADS group. Clearly the early and late risks involved with RYGB (or OADS), such as deep surgical site infections, intestinal complications, nutritional deficiencies [37], dumping syndrome [38] are greater than for HHR alone. These procedures are themselves associated with ongoing risk for further operative intervention [39] and in this cohort 24% of patients in the RYGB group went on to further surgery due to late complications at a mean of 20 months. This compares to 13.8% of HHR group who went on to further surgery at a mean of 25 months due to recurrence or persistence of reflux only, as there were no other late complications. Importantly, HHR does not preclude future additional anti-reflux procedures being performed such as RYGB or OADS should symptoms persist.

While this study demonstrates that HHR alone can adequately control reflux after LSG, workup and appropriate patient selection is paramount. In this cohort, patients who presented with PPI resistant reflux symptoms underwent upper endoscopy and 3D CT and/or manometry before discussing which therapeutic options may be open to them. Patients without significant weight regain, a sleeve stenosis resistant to pneumatic dilation to at least 30–35 mm and without esophageal motility disturbances were offered HHR as preferred surgical therapy, with RYGB or OADS surgery planned as a back-up if the patient was unhappy with their outcomes. OADS was offered to patients with weight regain without significant sleeve dilation or if a functional angularis stenosis was noted that could be corrected by straightening out the sleeve tube. An additional relevant factor related to patient preferences to be converted to RYGB or OADS rather than HHR, whereas others with some degree of esophageal dysfunction or sleeve asymmetry chose HHR over RYGB as a preference.

### Limitations

The retrospective nature of this study is a limitation, however sufficient data have been generated to create sufficient equipoise to justify an RCT in the future. We have a limited sample size, and while we identified a total of 99 eligible patients for late follow-up, over half were unable to be contacted or did not wish to participate. A further limitation is the highly selected nature of the patient cohort and the use of subjective primary outcomes to identify patients with symptomatic GERD via patient questionnaires, as opposed to demonstrating more objective follow-up evidence such as endoscopic or pH study findings of GERD. Ideally future studies would combine both subjective and objective assessment at both pre- and post-operative timepoints.

## Conclusion

This study demonstrates HHR (Cruroplasty with mesh reinforcement and esophagopexy) is a safe procedure that may result in equal efficacy and superior safety as compared to RYGB or OADS in the management of appropriately selected patients with medically refractory gastroesophageal reflux following LSG, warranting further studies to evaluate this approach.

## Data Availability

The datasets used and/or analysed during the current study available from the corresponding author on reasonable request.
